# Cellular and molecular mechanisms of neuronal loss in Atrx-knockout mice

**DOI:** 10.1186/1756-8935-6-S1-P42

**Published:** 2013-03-18

**Authors:** Pamela S Lagali, Chantal F Medina, Keqin Yan, Alan J Mears, Valerie A Wallace, David J Picketts

**Affiliations:** 1 Regenerative Medicine, Ottawa Hospital Research Institute, Ottawa, Ontario, Canada, K1H 8L6; 2 Vision Programs, Ottawa Hospital Research Institute, Ottawa, Ontario, Canada, K1H8L6; 3 Department of Biochemistry, Microbiology, Immunology, Faculty of Medicine, University of Ottawa, Ontario, Canada, K1H8M5; 4 Department of Ophthalmology, Faculty of Medicine, University of Ottawa, Ontario, Canada, K1H8M5; 5 University of Ottawa Eye Institute of The Ottawa Hospital, Ottawa, Ontario, Canada, K1H 8L6

## Background

Atrx is a member of the SNF2 family of chromatin remodeling proteins that functions by remodeling or repositioning nucleosomes at specific target genes using the energy from ATP hydrolysis. Mutations in the gene encoding Atrx cause the human ATR-X syndrome, an X-linked disorder that is associated with severe mental retardation. We have shown that targeted deletion of this gene in experimental mouse models results in the loss of neuronal cell populations in the central nervous system (CNS). Compromised neuronal survival in Atrx mutants may underlie the intellectual impairment and cognitive deficits observed in ATR-X syndrome. We have generated transgenic mice in which interneurons critical for modulation and integration of synaptic activity in the retina are selectively lost. We are using this model system to delineate the cellular and molecular mechanisms of neuronal cell loss in Atrx mutants.

## Objective

To determine the neuronal circuitry and genetic regulation underlying the loss of retinal interneurons in mice lacking the chromatin remodeling protein Atrx.

## Materials and methods

Conditional knockout approaches are used to selectively remove Atrx from different retinal cell populations *in vivo*, including the production of transgenic mice that express Cre recombinase embryonically and the ocular injection and electro-poration of Cre-expressing plasmids targeted to specific cell types postnatally. The phenotype of Atrx-deleted retinas is assessed by immunohistochemical analysis using antibodies recognizing retinal amacrine and horizontal cell marker proteins, including Pax6, syntaxin, choline acetyltransferase, and calbindin. Retinal function is examined by electroretinography. Gene expression changes are assessed with DNA microarrays and quantitative RT-PCR.

## Results

Retinal amacrine and horizontal cell disorganization and loss occurs when Atrx is deleted in multipotent progenitor cells early in retinal development using a Pax6alpha-Cre transgenic mouse driver line, but not when the gene is inactivated in lineage-restricted, post-mitotic amacrine and horizontal precursor cells targeted with a Ptf1a-Cre driver line. Electroretinograms show functional deficits in interneuron communication within the inner retina of the Pax6alpha-Cre-driven conditional knockout mice, suggesting a role for bipolar neurons in the observed phenotype. Deletion of Atrx postnatally through the injection and retinal expression of Cre-encoding plasmids recapitulates some features of the early knockout phenotype, further suggestive of a causative role played by later born neurons such as bipolar cells. In addition, genetic profiling of the mutant mice reveals misexpression of bipolar cell marker genes as well as genes encoding proteins that function in retinal synaptic communication.

## Conclusions

The loss of amacrine and horizontal cells from Atrx-deleted retinas appears to occur through a cell non-autonomous mechanism. Electrophysiological analysis, cell type-selective gene inactivation in the retina and gene expression profiling implicate a role for bipolar cells in mediating Atrx-dependent retinal inhibitory interneuron survival and function. Atrx may be involved in the regulation of specific genes that play a role in retinal neuron homeostasis, synaptic activity, and connectivity. These functions may extend to Atrx-mediated survival of neurons in other regions of the CNS.

